# Motility patterns of *Trypanosoma cruzi* trypomastigotes correlate with the efficiency of parasite invasion in vitro

**DOI:** 10.1038/s41598-020-72604-4

**Published:** 2020-09-28

**Authors:** Jorge A. Arias-del-Angel, Jesús Santana-Solano, Moisés Santillán, Rebeca G. Manning-Cela

**Affiliations:** 1 Unidad Monterrey, Centro de Investigación y de Estudios Avanzados del IPN, Apodaca, NL Mexico; 2 Depto. de Biomedicina Molecular, Centro de Investigación y de Estudios Avanzados del IPN, CDMX, Mexico City, Mexico

**Keywords:** Biophysics, Cell biology, Microbiology, Diseases, Pathogenesis

## Abstract

Numerous works have demonstrated that trypanosomatid motility is relevant for parasite replication and sensitivity. Nonetheless, although some findings indirectly suggest that motility also plays an important role during infection, this has not been extensively investigated. This work is aimed at partially filling this void for the case of *Trypanosoma cruzi*. After recording swimming *T. cruzi* trypomastigotes (CL Brener strain) and recovering their individual trajectories, we statistically analyzed parasite motility patterns. We did this with parasites that swim alone or above monolayer cultures of different cell lines. Our results indicate that *T. cruzi* trypomastigotes change their motility patterns when they are in the presence of mammalian cells, in a cell-line dependent manner. We further performed infection experiments in which each of the mammalian cell cultures were incubated for 2 h together with trypomastigotes, and measured the corresponding invasion efficiency. Not only this parameter varied from cell line to cell line, but it resulted to be positively correlated with the corresponding intensity of the motility pattern changes. Together, these results suggest that *T. cruzi* trypomastigotes are capable of sensing the presence of mammalian cells and of changing their motility patterns accordingly, and that this might increase their invasion efficiency.

## Introduction

Chagas disease (also known as American trypanosomiasis) is one of the neglected tropical diseases (NTDs) recognized by the World Health Organization^1^. Due to its etiology and its widespread distribution, it is one of the most important NTDs in Latin America. Over the last years, this disease has turned into a global health problem, given that its etiological agent (the protozoan parasite *Trypanosoma cruzi*) has expanded its distribution worldwide. It is estimated that about 6 to 7 million people are currently infected with this parasite^1^.

*Trypanosoma cruzi* exhibits a digenetic life cycle, alternating its residence between invertebrate and mammalian hosts. Along this cycle, *T. cruzi* goes through four different stages: **epimastigote**, **amastigote**, **metacyclic trypomastigote** and **bloodstream trypomastigote**. **Epimastigote** and **amastigote** are the replicative stages in the invertebrate and the mammalian hosts, respectively. Meanwhile, **trypomastigote** is regarded as the main infective stage^2^, and it is present in both the mammalian (bloodstream form) and the invertebrate hosts (metacyclic form). The parasite is flagellated in all stages. However, only epimastigotes and trypomastigotes have a long flagellum and are highly motile. Amastigotes have a truncated flagellum and are non-motile.

*Trypanosoma cruzi* trypomastigotes and epimastigotes are propelled by a single flagellum, via mechanochemical oscillations that generate motile forces. Other than motility, the flagellum of several microorganisms is also involved in functions like: sensitivity, cell division, morphogenesis, basal body migration, cytokinesis, cell signaling, host-microorganism interactions, and infection^3–6^. In kinetoplastids, flagellar motility participates in the parasite dissemination and annidation within the mammalian host, in the successful colonization of the insect vector, in different steps of the infection process, and in the clearance of antibodies from the parasite surface^7–11^. In *T. cruzi*, the truncated flagellum of intracellular amastigotes establishes a close contact with host mitochondria, and this interaction has been suggested to have a role in intracellular infection and parasite pathogenesis^12^. Recent studies have shown that various factors that affect the motility of *T. cruzi* trypomastigotes also inhibit infection^13,14^. Despite all these findings, the scientific literature aimed at studying the motility of *T. cruzi* is scarce^15^.

Taking the above considerations into account, the present work is aimed at improving our understanding of the role played by the motility of *T. cruzi* trypomastigotes during the infection process. The reasons for selecting this particular stage are that: trypomastigote is the motile stage of *T. cruzi* responsible for natural infection and, although all parasite stages are capable of establishing cell infection in vitro, trypomastigotes and amastigotes are more infective than epimastigotes^2^, while amastigotes are non-motile.

In infectivity assays carried out in mice with different *T. cruzi* strains, all the studied strains showed tropism for specific tissues, albeit to a different degree^16^. For instance, all the studied strains were myotropic, but they showed differences in the specific muscle tissues that they preferably invaded. A possible explanation for this observation is that *T. cruzi* parasites invade some cell types more efficiently than others. Thus, under the assumptions that this is true and that trypomastigote motility is involved in the infection process, we pose the following hypothesis: *T. cruzi* trypomastigotes are capable of sensing the presence of in-vitro-cultured mammalian cell lines, they alter their motility patterns accordingly, the magnitude of the change depends on the cell line, and this is correlated with the efficiency with which the parasite invades such cell line. In the following sections, we present the methodology we devised to test this hypothesis, as well as the obtained results, discussion and conclusions.

## Results

### Motility assays

**Figure 1:**
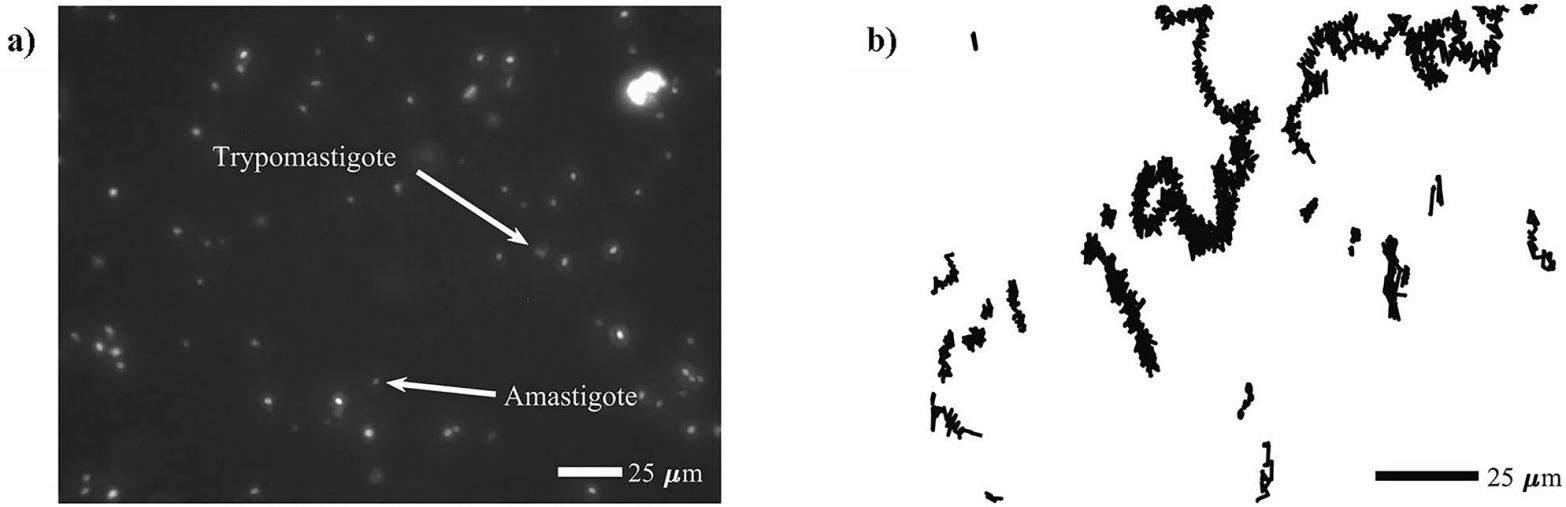
(**a**) Typical frame from a recorded video. Amastigotes and trypomastigotes can be easily identified via fluorescence microscopy because the parasites have been transfected with the coding sequence of GFP. (**b**) Reconstructed trypomastigote trajectories, which can either be quite long, or highly confined.

We started by characterizing the motility patterns of *T. cruzi* trypomastigotes under control conditions. That is, when only parasites and no mammalian cells are present in the medium. We followed the methodology described in the Materials and Methods section to record the swimming of parasites (in the microfluidics devices that we designed and built to that purpose), and to recover individual trypomastigote trajectories. In this regard, it is important to mention that the employed video resolution (640x480 pixels), combined with the microscope magnification (40X), were enough to differentiate between trypomastigotes and amastigotes (as some contaminant amastigotes were present in the medium and both of them were visible using fluorescence microscopy)—see Fig. 1a. The spatial resolution corresponding to these experimental conditions was $2.52 \, \hbox {pixels}/\upmu \hbox {m}$. We computed the average instantaneous speed of all the recovered parasite trajectories, and noticed that the average speed of amastigote trajectories never goes above $4 \, \upmu \hbox {m/s}$. Thus, we used this threshold value as a second filter to discard those trajectories that do not correspond to trypomastigotes. Typical recorded trypomastigote trajectories are shown in Fig. 1b.

Once we made sure of having only trypomastigote trajectories, we characterized them by computing their mean speed values and their mean squared displacements as explained below. Let us represent a trajectory by the arrays:

$$\begin{aligned} t_i, \quad x_i, \quad y_i, \end{aligned}$$

where variables *t*, *x*, and *y* respectively denote time, the horizontal coordinate, and the vertical coordinate, while subscript *i* refers to the frame number in the video. Since the videos were taken at 50 frames per second,

$$\begin{aligned} \Delta t = t_{i + 1} - t_i = 0.02 \, \text {s}, \end{aligned}$$

for all values of *i*. With these definitions, the mean parasite speed along a single trajectory consisting of *N* points is:

$$\begin{aligned} {\overline{v}} = \frac{1}{N-1} \sum _{i=1}^{N-1} \frac{\sqrt{(x_{i+1} - x_i)^2 + (y_{i+1} - y_i)^2}}{\Delta t}. \end{aligned}$$

On the other hand, the mean squared displacement (MSD) can be computed as follows for a trajectory consisting of *N* points:

$$\begin{aligned} \text {MSD}(t_j) = \frac{1}{N-j} \sum _{i=1}^{N-j} (x_{i+j} - x_i)^2 + (y_{i+j} - y_i)^2, \end{aligned}$$

where $t_j = j \Delta t$. From its definition, MSD measures the deviation of the position of a randomly moving particle with respect to a reference position over time, and it can be thought of as measuring the area explored by such particle. In general, the mean squared displacement obeys a power-law of the form $\text {MSD}(t) \propto t^{\lambda }$ for short periods of time. The value of $\lambda$ determines whether the motion is subdiffusive ($\lambda < 1$), diffusive ($\lambda =1$), or superdiffusive ($\lambda >1$).

We performed 5 independent experiments, and recorded a total of 15 (2.5 min long) videos (3 per experiment). From these videos, we recovered 3,934 independent trajectories, with an average of 290 points per trajectory. We statistically analyzed the trajectory features for each of independent experiment, and found no significative difference among them. Thus, we grouped together all of the trajectories obtained under control conditions to improve the statistics. According to our results, the averaged trajectory mean speed, $\langle {\overline{v}} \rangle$, and MSD power-law exponent, $\langle \lambda \rangle$ (which was computed by considering the MSD values at times in the range 0–2 s), take the following values:

$$\begin{aligned} \langle {\overline{v}} \rangle = 8.1258 \pm 0.48 \upmu \text {m/s}, \quad \langle \lambda \rangle = 1.004 \pm 0.004. \end{aligned}$$

After characterizing trypomastigote motility under control conditions, we performed experiments in which the parasites swim above monolayers of in vitro-cultured mammalian cell lines. As described in the methodology, we made use of four different cell lines, and considered three different confluences: low confluence (LC), $\sim$25%; medium confluence (MC), $\sim$50%; and high confluence (HC), $\sim$100%. For each condition, we performed up to 5 independent experiments, recorded 3 videos per experiment, and recovered a similar number of trajectories per video as in the control experiments. We characterized the recovered trajectories by computing the average mean speed and the average MSD power-law exponent. Below, we discuss the obtained results.

**Figure 2:**
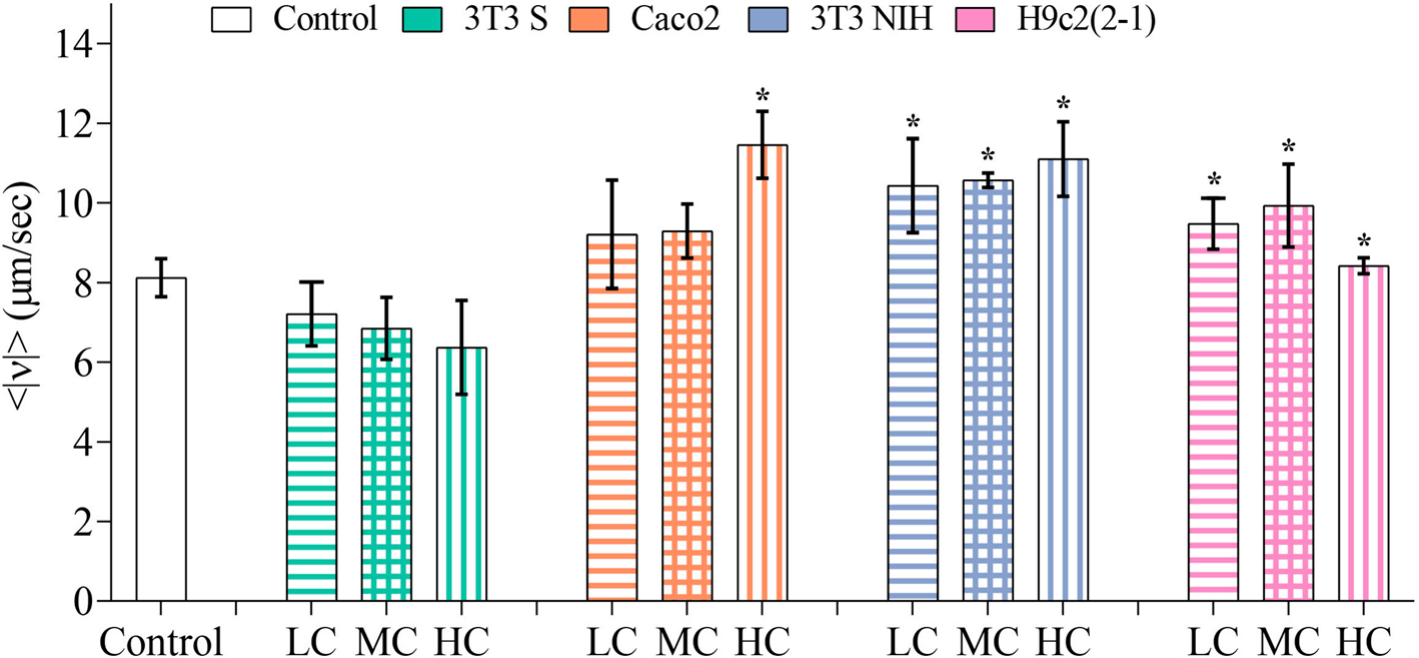
Average mean speed values of trypomastigotes interacting with different mammalian cell lines. The number of independent experiments performed for each of the experimental conditions is as follows: control group, n = 5; 3T3 S, n = [4 (LC), 3 (MC), 3 (HC)]; Caco2, n = [3 (LC), 3 (MC), 3 (HC)]; 3T3 NIH, n = [3 (LC), 3 (MC), 3 (HC)]; and H9c2(2-1), n = [4 (LC), 3 (MC), 4 (HC)]. LC, MC, and HC respectively stand for low, medium, and high confluence. Error bars indicate 95% confidence intervals. Asterisks denote statistically significant, based on confidence intervals.

In Fig. 2 the average mean speed values obtained for all of the experimental and control conditions are contrasted. Observe that, when trypomastigotes swim above monolayers of Caco2, 3T3 NIH, and H9c2(2-1) cells, they move faster than in the control group. Conversely, their speed shows a slight decrease in the presence of 3T3 S cells. In the case of 3T3 S, Caco2 and 3T3 NIH cells, we found no dependence of the parasite mean speed on the cell confluence. For H9c2(2-1) cells, our results indicate that the parasite mean speed decreases at high confluence. However, this could be caused by myoblasts differentiation into myotubes at $\sim$100% confluence^17^.

**Figure 3:**
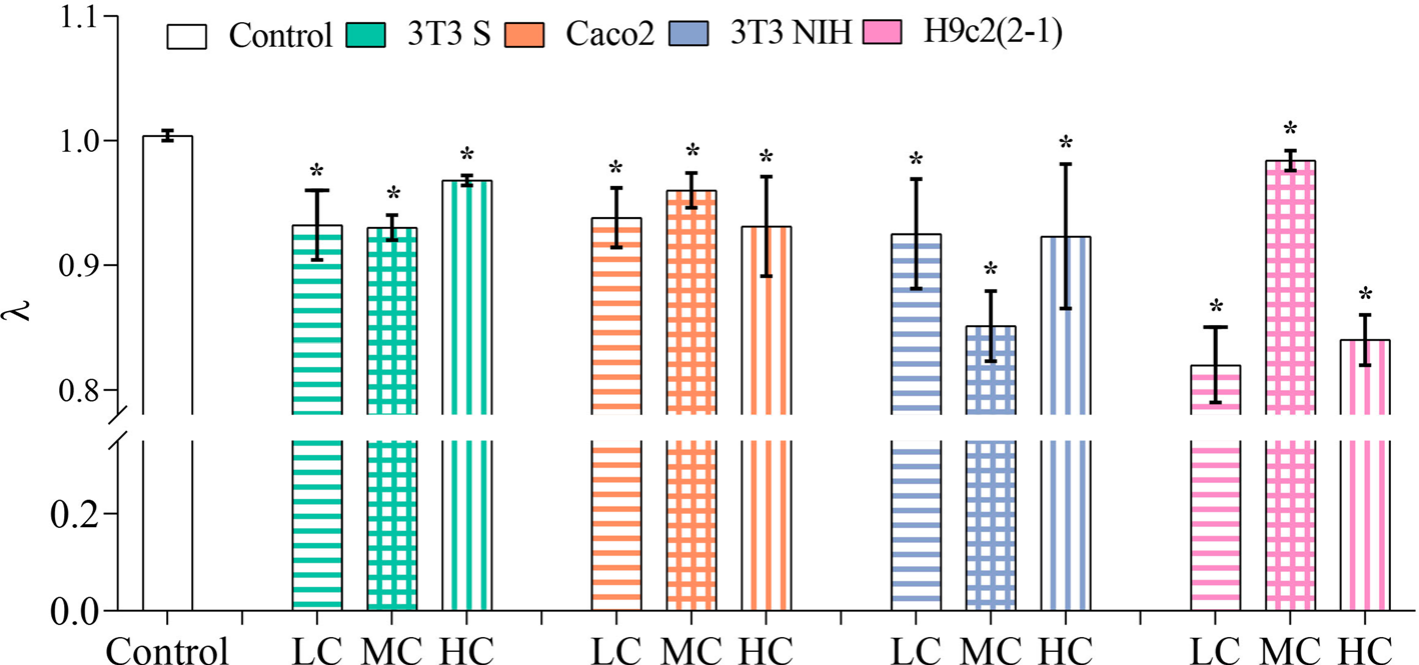
$\lambda$ values obtained from a linear regression of the MSD curves ($t^{-1}$). The number of independent experiments performed for each of the experimental conditions is as follows: control group, n = 5; 3T3 S, n = [4 (LC), 3 (MC), 3 (HC)]; Caco2, n = [3 (LC), 3 (MC), 3 (HC)]; 3T3 NIH, n = [3 (LC), 3 (MC), 3 (HC)]; and H9c2(2-1), n = [4 (LC), 3 (MC), 4 (HC)]. LC, MC, and HC respectively stand for low, medium, and high confluence. Error bars indicate 95% confidence intervals. Asterisks denote statistically significant, based on confidence intervals.

The obtained results for the MSD power-law exponent ($\lambda$) are shown in Fig. 3. Notice that, in all the experimental conditions, the value of $\lambda$ is consistently smaller than under control conditions. This means that, in the presence of mammalian cells, trypomastigotes tend to move in a subdiffusive fashion in short periods of time ($\le 2 \, \hbox {s}$), while under control conditions their motion is approximately diffusive.

Recalling the meaning of the mean squared displacement, the results in the above paragraph reveal that *T. cruzi* trypomastigotes spread out from an initial position more slowly when they are in the presence of mammalian cells (Fig. 3). Once again, the value of $\lambda$ resulted to be independent of cell confluence in the case of 3T3 S, Caco2 and 3T3 NIH cells. The value of $\lambda$ decreased in the presence of H9c2(2-1) cells at $\sim$100% confluence (this may be a result of cell differentiation into myotubes^17^), as well as at low confluence (we do not have a plausible explanation for this observation).

**Figure 4:**
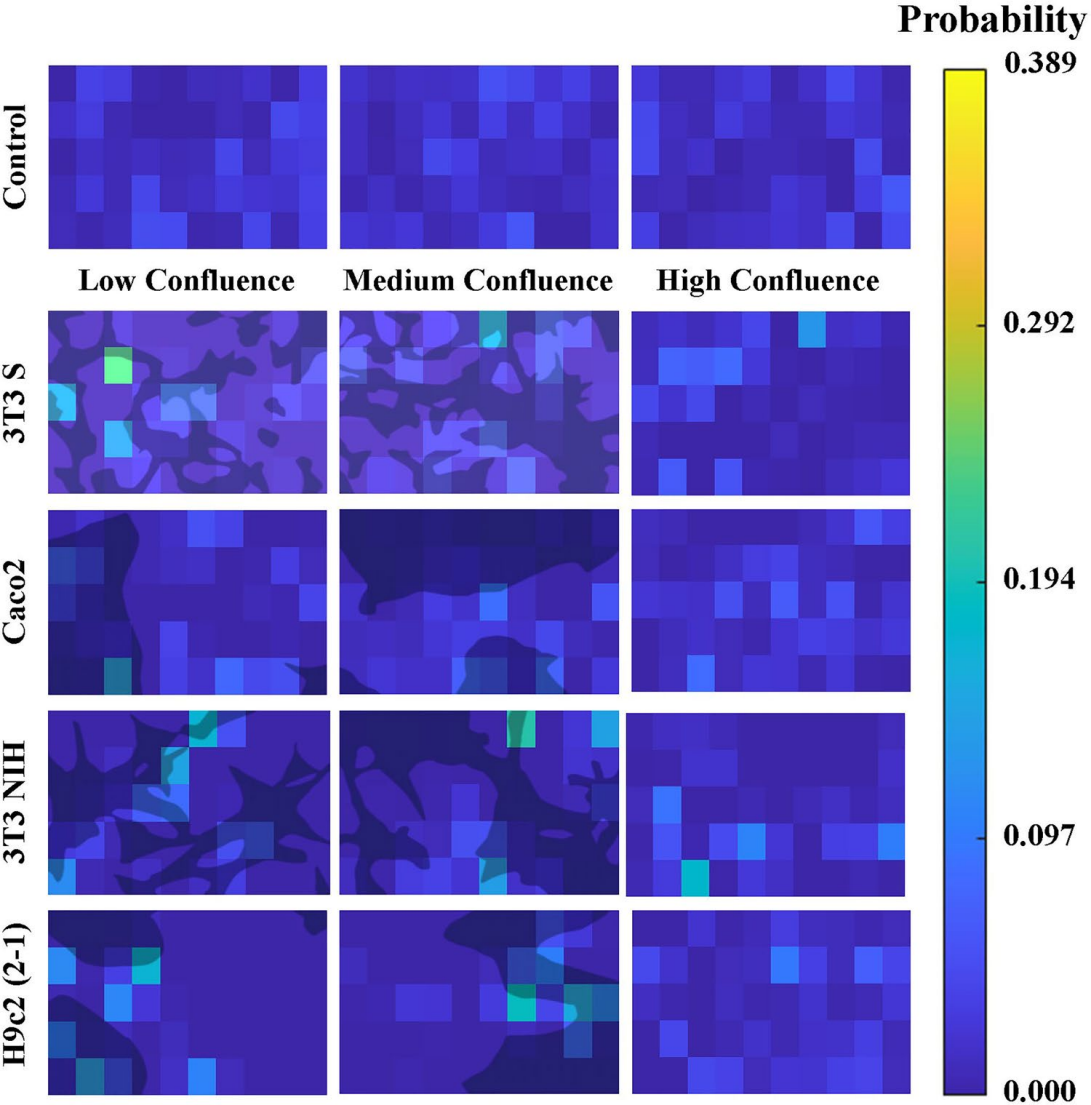
Spatial probability-distribution maps. Upper row: control-group representative maps. The influence of 3T3 S, Caco2, 3T3 NIH and H9c2 (2-1) cells on the spatial distribution of trypomastigotes is illustrated in the images on the second, third, fourth and fifth rows, respectively. For the experimental group maps, the first, second and third columns respectively corresponds to low ($\sim$25%), medium ($\sim$50%), and high ($\sim$100%) confluence experiments. Dark-shaded areas correspond to the surface occupied by the cells in the experiments of low and medium confluence. No dark-shaded areas are shown in the third column maps because, in the high-confluence experiments, the entire field is covered by cells. This figure was elaborated employing the software MATLAB R2018a/URL: https://www.mathworks.com/products/matlab.html.

The observed $\lambda$-value decrease could be an indication of the parasite tendency to move in restricted areas when mammalian cells are present. To test this, we analyzed each one of the recorded videos as follows. First, we identified the position of all the trypomastigotes in each video-frame. Then, we lumped together the parasite positions recovered from all frames into a single image. Finally, we divided the image surface into 50 equally-sized rectangles, and computed the fraction of position points lying in every rectangle. These fractions can be interpreted as the probability distribution of finding a parasite in different positions across the recorded surface. Representative plots of the resulting probability distributions, obtained under different experimental conditions, are shown in Fig. 4. We can appreciate there that the distribution of position points is homogeneous in the absence of mammalian cells. However, when mammalian cells are present, spots with high concentrations of data points appear.

**Figure 5:**
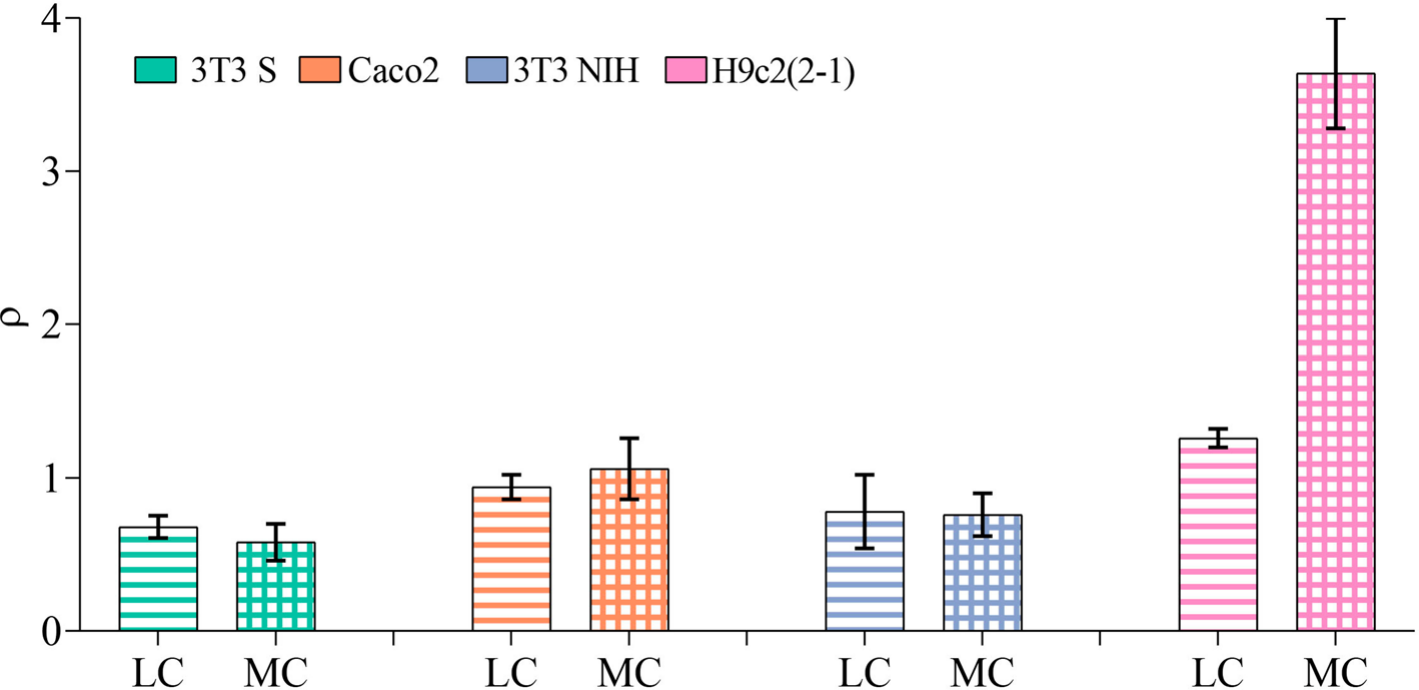
$\rho$ values from experiments performed under low and medium cell-confluence conditions. Error bars indicates the 95% confidence intervals.

We can also notice in Fig. 4 that the spots with high concentration of position points tend to be over or nearby mammalian cells. To quantitatively analyze this observation, we computed the probability per unit area (or probability density) that the parasite trajectory points lie above or in the vicinity (within $4 \, \upmu \hbox {m}$ of their periphery) of mammalian cell profiles, $P_{\text {ON}}$, as well as the probability density that the trajectory points lie on empty space, $P_{\text {OFF}}$. Then, we computed the ratio $\rho = P_{\text {ON}} / P_{\text {OFF}}$. Values of $\rho$ larger than one indicate that it is more likely that the parasites locate above or in the vicinity of mammalian cells than away from them. The results obtained for all cell lines and experimental conditions are shown in Fig. 5. Notice that, in the case of Caco2 and 3T3 NIH cells, the parasites show no preference to locate nearby the cells or away from them. In the presence of 3T3 S cells, the parasites seem to prefer being away from the cells. Finally, they clearly prefer to be above or nearby H9c2(2-1) cells.

Summarizing, the results presented above demonstrate that *T. cruzi* trypomastigotes alter their motility characteristics (speed, MSD power-law exponent, and localization) when mammalian cells are present, and that the extent of these changes depend on the cell line.

### Infectivity assays

**Figure 6:**
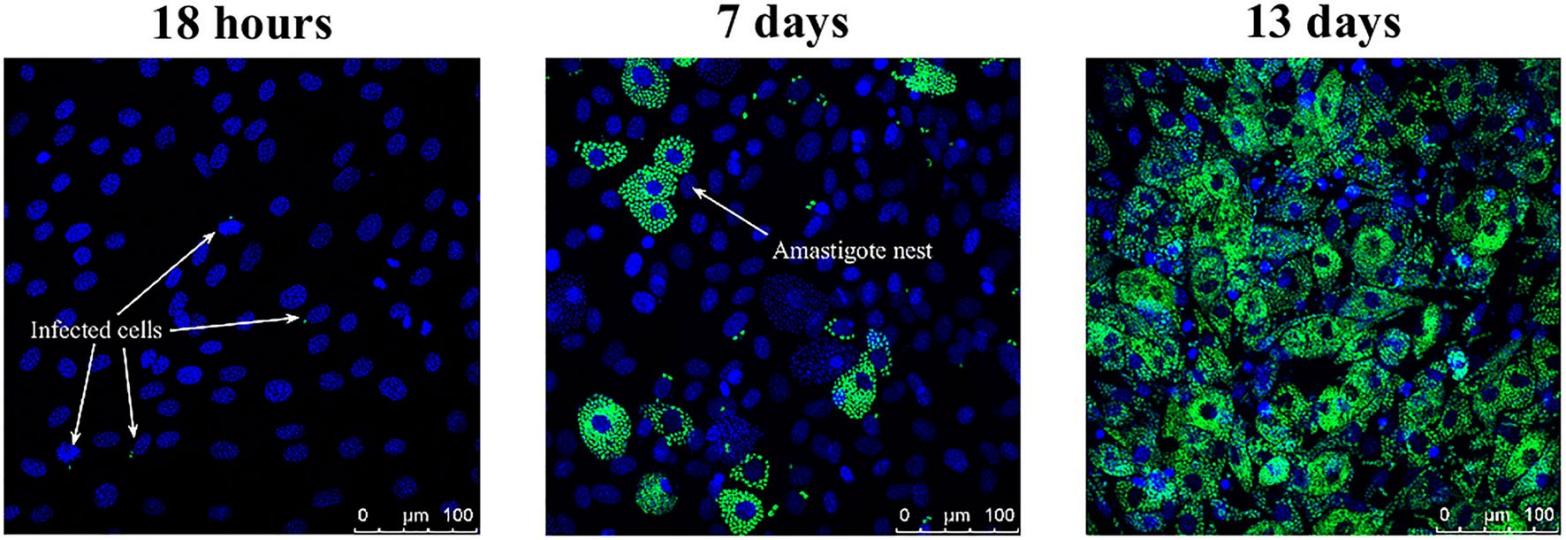
Representative images of a CL Brener trypomastigotes infection over 3T3 NIH cells: *left*, after 18 hours of the initial interaction; *medium*, after 7 days; and *right*, after 13 days. Cell nucleus, but also parasites nucleus and kinetoplast are dyed in blue, while parasites are identified by the presence of the GFP protein.

We started by studying the infection progression in 3T3 NIH cells. Trypomastigotes were added to the cell culture (as detailed in the Materials and Methods section) when it had a $\sim$50% confluence, and the evolution of the infection was monitored by means of fluorescence microscopy. A typical sample of the obtained results is presented in Fig. 6. There, we can appreciate that, after 18 h of the initial cell-parasite interaction, a few cells had already been infected. Since amastigote is the obligated intracellular stage of the parasite, an infected cell can be identified via the presence of at least one amastigote (a spherical green spot) in the neighborhood of the cell nucleus^12^. As the infection progresses, the number of infected cells increases, as well as the number of amastigotes per infected cell. After 13 days of interaction, almost all the cells have been infected. From these results, we concluded that the fraction of infected cells after 18 h of the initial cell-parasite interaction can be used as an estimate for the parasite invasion efficiency, in agreement with former results^12,18–20^.

**Figure 7:**
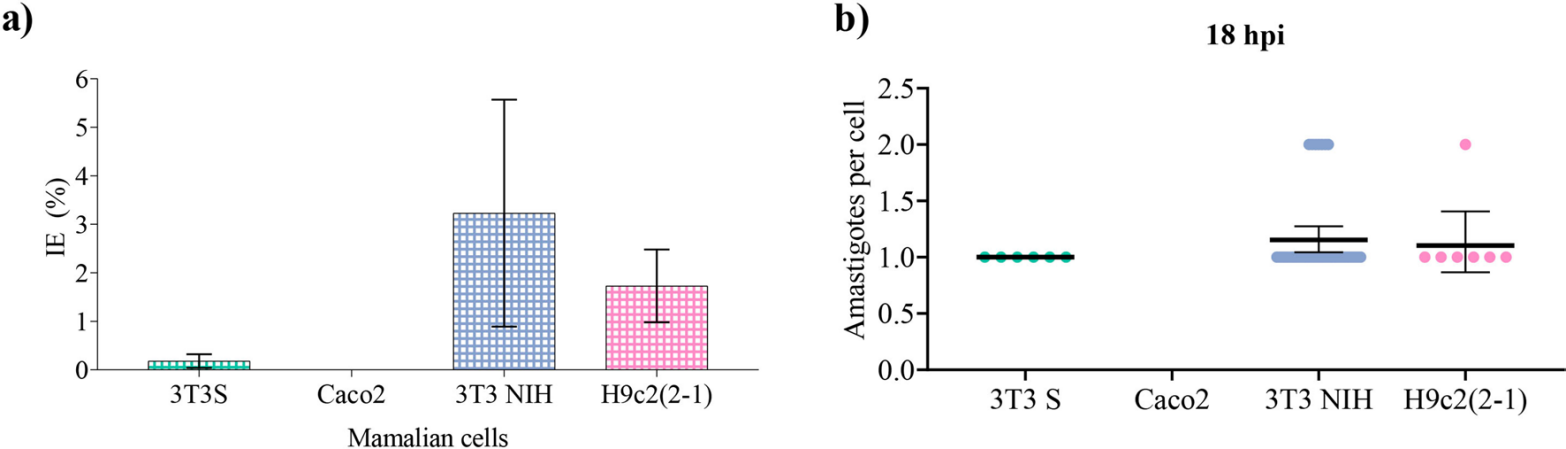
(**a**) Trypomastigote invasion efficiency (IE) for the different studied cell lines. Error bars represent 95% confidence intervals obtained from 3–4 independent experiments. (**b**) Number of amastigotes per infected cell at 18 h post interaction. Bold horizontal lines corresponds to the average number of parasites per cell. Error bars indicate 95% confidence interval.

After establishing the protocol, we performed infectivity assays for all the studied mammalian cell lines and measured the corresponding invasion efficiency. The obtained results are reported in Fig. 7a. Observe that the invasion efficiency for the 3T3 S and Caco2 cell lines is almost negligible as compared to that of 3T3 NIH and H9c2 (2-1) cells. To our consideration, this demonstrates that the infection susceptibility is cell specific. To further support this finding, we measured the number of amastigotes per infected cell (APC) at 18 h post interaction (hpi) (Fig. 7b). We measured a global average of 1.4 APC for the four studied cell lines, indicating that the infection had just begun. Only in the cases of 3T3 NIH fibroblasts and H9c2(2-1) myoblasts, we could identify more than one (but never more than two) APC (Fig. 7b), meaning that amastigotes have started to replicate. Remarkably, we could not find any infected Caco2 cell.

### Correlation between motility changes and invasion efficiency

We have seen that CL Brener *T. cruzi* trypomastigotes change their motility patterns in the presence of mammalian cells; and that the extent of these changes depends on the type of cell. On the other hand, the same parasites invade the studied mammalian cell types with a differential efficiency. These results prompted us to investigate a possible correlation between motility and invasion efficiency.

**Figure 8:**
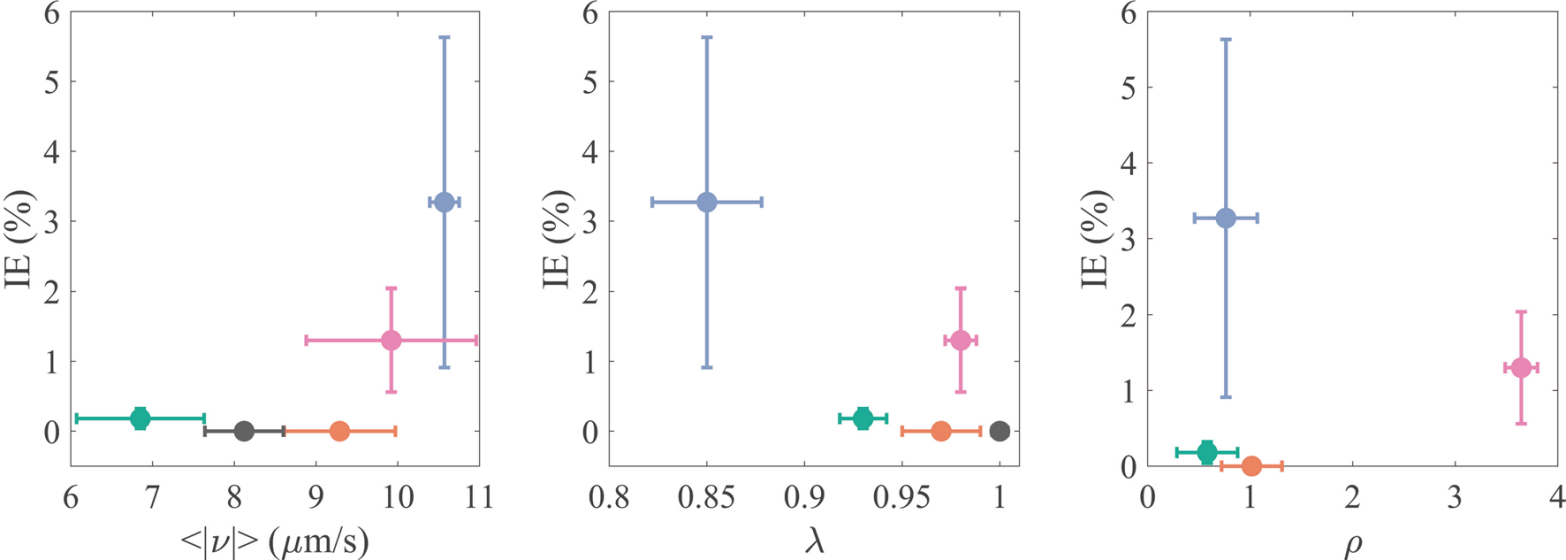
Scatter plots in which the invasion efficiency is contrasted to the parameters that characterize trypomastigote motility. The color code is as follows for the different experimental conditions: control (gray), 3T3 S (green), Caco2 (orange), 3T3 NIH (blue) and H9c2(2-1) (pink). Error bars indicate the 95% confidence interval for both axes.

In Fig. 8, we present scatter plots where the invasion efficiency for the four analyzed cell types is contrasted with the parasite motility characteristics in the presence of the same cell types. Since the infectivity assays were performed with a confluence of about 50%, we used the motility assay results obtained with the same confluence. Observe that the invasion efficiency is positively correlated with the average parasite mean speed, and negatively correlated with the MSD power-law exponent. No clear correlation was found between the invasion efficiency and the probability that the parasites are located above or nearby the cells they infect. These results suggest that the motility modifications undergone by the parasites in the presence of mammalian cells may be functionally related to the cell invasion process.

## Discussion and conclusions

We have progressed a long way in unveiling the basic properties of flagellum dynamics and cell motility of *T. cruzi* in culture media^15,21^. However, culture-medium microenvironmental conditions greatly differ from those present in mammalian hosts. We proved in a former work that epimastigotes, the culture forms of *T. cruzi*, swim in the direction of their flagellum, due to tip-to-base symmetrical flagellar beats. This flagellum beating mode is frequently interrupted by base-to-tip highly asymmetric beats. Switching between both beating modes facilitates parasite reorientation, thus allowing a large repertoire of movements and trajectories^15^. We further characterized epimastigote motility by analyzing recorded trajectory-step features such as: step length, angular change of direction, longitudinal and transverse displacements with respect to the previous step, and mean square displacement. We developed a mathematical model to simulate parasite trajectories and better understand its motility^21^. Our results revealed that epimastigote motility is characterized by alternated run and tumbling periods, which result in the alternation of quasi-rectilinear and highly confined and intricate paths^15^. While these works have improved our understanding of *T. cruzi* epimastigote motility, our knowledge of *T. cruzi* trypomastigote motility is still scarce and, as far as we know, its relevance in the natural host microenvironment has not been deeply investigated.

In the present work, we have characterized the motility of *T. cruzi* trypomastigotes (CL Brener strain) and investigated how the parasite motility patterns change in the presence of in vitro-cultured mammalian cell lines. We have also quantified the efficiency with which the parasites invade the same cell lines, and found that the average parasite mean speed and MSD power-law exponent are correlated (positively and negatively, respectively) with the invasion efficiency. These results agree with the notion that motility might play an important role during the infection process, although this possibility is yet to be determined.

It is illustrative to compare our findings with those of other works on *T. cruzi* and other similar parasites. For instance, the mean speed value we found for free-swimming CL Brener *T. cruzi* trypomastigotes—${\sim }8.12 \, \upmu \hbox {m/s}$—is of the same order of magnitude as that of Lister 427 *T. brucei*—${\sim }8.9 \, \upmu \hbox {m/s}$—^22^, and EATRO-427 *T. brucei* trypomastigotes—${\sim }2 \, \upmu \hbox {m/s}$—^23^. However we must be cautious while making this comparison because parasites swim in the bulk in our experimental setup, whereas in the works reported in^22^ and^23^, they are constrained to move within a quasi-2-dimensional space. The fact that the average swimming speed of *T. brucei* trypomastigotes seems to be strain dependent, prompts us to extend our experiments using different strains, not only to characterize their motility, but also to investigate how it is related with their invasion efficiency (see below). The trypomastigote average speed here reported is also similar to that previously reported for *T. cruzi* epimastigotes^21^—${\sim } 4 \, \upmu \hbox {m/s}$. However, we must take into consideration that epimastigotes were restricted to swim in a quasi-2-dimensional space in the last experiment, which impedes a straightforward comparison with our results.

Interestingly, *T. cruzi* epimastigotes^21^, as well as *T. brucei* trypomastigotes^22,23^ exhibited a superdiffusive movement at short time scales (of the order of a few seconds). Contrarily, we found that free-swimming CL Brener *T. cruzi* trypomastigotes display a diffusive motility pattern. This could indicate that the parasites we study have a higher degree of confinement than those analyzed in previous works^21–23^. However, the different experimental conditions cannot be ignored. While, in our experimental setup, parasites swim in the bulk, they swim very close to rigid surfaces, on top and below, in the experiments reported in^21–23^, and hydrodynamic interactions with those surfaces may alter the parasite motile behavior^24^.

We have found that CL Brener *T. cruzi* trypomastigotes modify their motile behavior in the presence of mammalian cell lines. In general, the parasites increase their mean speed, they tend to explore smaller areas at short time scales, and (in some cases) show a preference to be located on top or nearby cells’ periphery. Notably, the extend of these changes depends on the cell type. These results are consistent with the possibility that *T. cruzi* trypomastigotes are capable of sensing mammalian cells, and in consequence modifying their motility patterns; probably, to prepare themselves for infection.

Different microorganisms sense the host environment to trigger molecules and signaling cascades, that ensure an efficient infection and survival within host cells^25–28^. For instance, *Toxoplasma gondii* efficiently senses the environment to regulate the exocytosis of its Microneme, which crucially participates in the egress, gliding motility and invasion during the parasite lytic cycle^26^. In addition to its function in motility, the trypanosome flagellum appears to serve as a sensory organelle that could regulate parasite virulence, motility and cell-to-cell communication, among other processes^29–34^. In fact, it has been proposed that the microenvironment determines the *T. brucei* flagellum beating direction, as well as the parasite path^10^. With this perspective in mind, we performed infectivity assays with *T. cruzi* trypomastigotes and measured the corresponding invasion efficiency and the evolution of amastigote count per cell. We observed that the invasion efficiency and amastigote count per infected cell varied among cell lines, and were positively correlated with the extend of motility-pattern changes.

*T. cruzi* is able to infect a wide variety of nonprofessional phagocytic cells^18^, both in vitro^2,35–38^ and in vivo^39–42^. Although in the vertebrate host, the parasite can invade somatic cells in a wide range of tissues^43^, disease pathology occurs mostly in the heart and the digestive system. Moreover, different parasite strains infect different tissues with a variable efficiency^43^, due to the high genetic and phenotypic variability found among *T. cruzi* strains^44^. In this context, and taking into consideration the present work results, it would not be strange that *T. cruzi* senses the different signals of host tissues, and responds differently, not only in regard to its motile behavior, but also in the activation of enzymes and signaling pathways necessary for infection, survival, and intracellular replication. From this, we believe that the motility and infection analysis we have performed provide a useful platform for future cellular^13,14^ and genetic^11,21,45^ research, that would improve our understanding of the role played by parasites motility in the infection process.

In conclusion, our results suggest that *T. cruzi* trypomastigotes are capable of sensing mammalian cells (albeit to a different degree, depending on the cell type), and could modify their motility patterns to increase their invasion efficiency. Validating this hypothesis in future research and finding the involved mechanisms, would be not only interesting, but could also have biomedical implications.

## Methods

### Cell cultures

3T3 NIH embryonic mouse fibroblasts (ATCC CRL-1658), 3T3 Swiss-Albino (3T3-S) embryonic mouse fibroblasts (ATCC CCL-92), H9c2(2-1) rat myoblasts (ATCC CRL-1446), and Caco2 human colon epithelial (ATCC HTB-37) cells were grown in Dulbecco’s Modified Eagle Medium (DMEM), supplemented with 10% fetal bovine serum (FBS) and 0.5% penicillin/streptomycin ($100 \, \upmu \hbox {l/ml}$ penicillin/streptomycin), at $37^{\circ }\, \hbox {C}$ in an atmosphere of 5% ${\hbox {CO}}_{2}$. We have decided to work with these cell lines since they have shown different susceptibility to *T. cruzi* infection^2,35–38^ and are related with organs where disease pathology is develop^46^.

### Parasite cultures

Fluorescent epimastigotes from CL Brener strain were maintained in liver infusion tryptose (LIT) medium, supplemented with 10% fetal bovine serum (FBS), 0.5% penicillin (10,000 IU)/streptomycin ($10.000 \, \upmu \hbox {g}$), and 1% hemin (5 mg/ml), at $28^{\circ }\, \hbox {C}$. These stable transfected parasites were obtained in a previous work by electroporation with pTREXn-GFP DNA^2^. Cell-culture-derived trypomastigotes (CCDT) were obtained from supernatant of 3T3 NIH fibroblast monolayers infected with GFP-transfected parasites, as described in subsection Infectivity assays.

### Motility assays

Swimming CCDTs were recorded and their motility patterns were analyzed in the absence (control group) or presence of one of the above mentioned cells lines (experimental groups), which were grown at $\sim$25%, $\sim$50% and $\sim$100% confluence.

**Figure 9:**
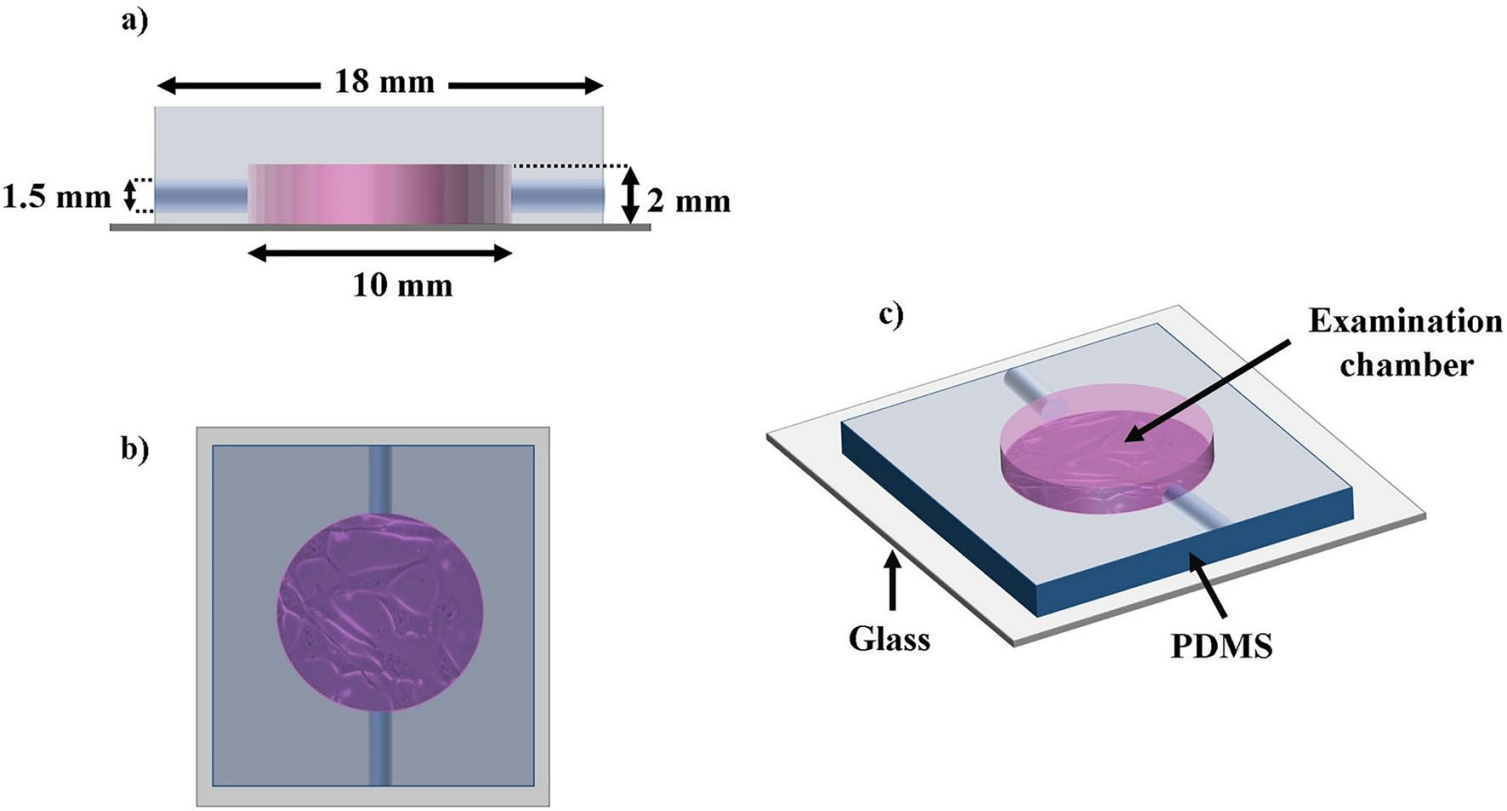
Lateral (**a**), superior (**b**), and perspective (**c**) views of the device designed and manufactured to study parasite motility. The examination chamber consist on a cylinder of 1 cm of diameter and 2 mm of height. This chamber has two perforations of 1.5 mm of diameter which where used as input and outlet of the sample. Figure designed and drawn by Jorge A. Arias-del-Angel.

Devices of Polydimethyl siloxane (PDMS) were fabricated to examine parasite motility in the absence of evaporation and flows (see Fig. 9). To this end, PDMS and its catalyst were mixed on a 1:10 proportion, and polymerized at $80^{\circ }\, \hbox {C}$ for 3 hours, on top on an acrylic mold in which cylindrical posts ($D = 10 \, \hbox {mm}$, $H = 2 \, \hbox {mm}$) have been manufactured with a micro milling machine (these pots leave holes in the PDMS waffles which were later used as examination chambers). After polymerization, the PDMS waffles were gently removed from the mold, and inlet and outlet channels were drilled on opposite sides of the examination chambers. The waffles were then washed in a sonicator, twice with MiliQ water for 30 minutes, once with isopropyl alcohol at 97% for 15 minutes, and once more with MiliQ water for 15 minutes. The waffles were then dried with nitrogen and they were kept in a dry and dustless place for at least 24 hrs. After that, the waffles were washed with PVP40 0.04%, and they were fixed to coverslips (previously washed with methanol) by means of a plasma sealer. The devices thus manufactured were wrapped with aluminum foil and autoclaved for 1 hour.

Cell-culture-derived trypomastigotes were harvested from primary infections by centrifuging at 1,048 g for 10 minutes in a HERMLE clinic centrifuge. Harvested parasites were suspended in 1 ml DMEM plus 2% FBS, and counted in a Neubauer chamber. Then, $4.5 {\times } 10^{4}$ trypomastigotes were taken into a fresh solution of DMEM plus 2% FBS, together with $1.35 {\times } 10^{5} \, 0.5 \, \upmu \hbox {m}$ diameter polymer particles (Fluoro-Max green fluorescent polymer micro spheres, Cat.no. G500). The final volume used per chamber was of $160\hbox {-}180 \, \upmu \hbox {m}$. The statistical properties of the particle trajectories were used as an exclusion criteria to discard those devices with flows inside the examination chamber.

In control experiments, the output channel of the examination chamber was connected to a 1.5 mm diameter TYGON tube, while the trypomastigote/particle solution was introduced through the input channel by means of a micropipette. Once the device was completely full, it was sealed. The device was then kept at $37^{\circ }\, \hbox {C}$ and 5% CO2 for 1 hr.

In the experimental groups, different cell inoculums were introduced into the examination chamber prior to the addition of parasites. After introducing the cells, they were allowed to grow in fresh medium, at $37^{\circ }\, \hbox {C}$ and 5% CO2, until they reached a $\sim$25%, $\sim$50% or $\sim$100% confluence. Then, the parasites were introduced as in control experiments. To measure cell confluence, we employed bright-field pictures of the cells, which were later binarized by means of Power Point tools. Then, the confluence was computed as the ratio of pixels corresponding to cells to the total pixels in the image.

Videos were recorded, at 50 frames per second, with a CCD camera in the middle of the examination chambers (to diminish the effect of the chamber walls on the parasite motile behavior), by means of an inverted phase contrast and fluorescence microscope (Olympus IX50), with a 40X magnification objective (640x480 pixels). The focal plane was fixed about $2\hbox {-}5 \, \upmu \hbox {m}$ over the cells. The temperature during the recording was controlled in the $36\hbox {-}38^{\circ }\, \hbox {C}$ range.

The trajectories of trypomastigotes, as well as those of the added polymer particles, were recovered by means of a custom software implemented in MATLAB^21^. The trajectories were then statistically analyzed by computing parameters such as the mean speed, the mean square displacement and the spatial distribution. In particular, the results of the polymer-particle-trajectory analysis were used to discard all those experiments in which a flux was detected.

### Infectivity assays

We performed primary infections as previously described by Manning and collaborators^47^. Monolayers of 3T3 NIH fibroblasts at 50% confluence were infected with $2 \times 10^{6}$ mid-log-phase florescent epimastigotes per ml of culture medium. Forty-eight hours later, the cells were washed with DMEM medium to remove non-adherent parasites, and fresh DMEM plus 2% FBS was added. The culture medium was replaced every other day. Cell-culture-derived trypomastigotes from this primary infection were recovered from supernatants and used for the motility assays described before, or to perform the infection kinetics experiments. To avoid the presence of amastigotes, only the first released CCDTs were used in all the experiments. For the secondary infections, $1.95 \times 10^{5}$ (1-3 parasites per cell) fluorescent CCDTs, obtained from primary infections, were used to infect 3T3 NIH, 3T3-S, H9c2(2-1) and Caco2 cells grown on coverslips at 50% confluence. After 2 h of cell-parasite interaction, the cultures were washed with DMEM until no parasites were attached. Coverslips were recovered at 18 h for invasion efficiency experiments for all four cell lines (or at different times, for 3T3 NIH cells) and then washed with 1X PBS, fixed with formaldehyde at 3.7% for 20 minutes at room temperature, washed again with 1X PBS, and finally mounted and stained with Vectashield with DAPI (*Vector*, Cat. 1500). The samples were analyzed with a LEICA confocal microscope, with a 40X oil-immersion objective (NO 24) and the fluorescence images captured and analyzed using the software MATLAB. As the trypomastigotes form was used to initiate the infections, a host cell was considered infected if amastigotes were observed inside the cell. The percentage of infected cells was calculated by comparing the number of cells containing parasites to the total number of cells, as well as the number of amastigotes per cell.

### Statistical analysis

The values of all the experimentally measured quantities is reported as: $\mu \pm 2 \sigma / \sqrt{N}$, where $\mu$ is the mean value, $\sigma$ is the standard deviation, and *N* is the number of independent experiments. The quantity $\sigma / \sqrt{N}$ is the standard error. Thus, $\mu \pm 2 \sigma / \sqrt{N}$ determines a 95% confidence interval.

The error bars in all the graphics correspond to 95% confidence intervals. We consider that the mean values of two experimental groups are different, with a confidence of 95%, when the corresponding intervals do not intersect. When this occurs, it is denoted with an asterisk.
